# Soft Robotics for Parkinson’s Disease Supported by Functional Materials and Artificial Intelligence

**DOI:** 10.34133/bmef.0143

**Published:** 2025-07-02

**Authors:** Hirak Mazumdar, Kamil Reza Khondakar, Suparna Das, Ajeet Kaushik

**Affiliations:** ^1^ Department of Computer Science and Engineering, Adamas University, Kolkata, West Bengal 700126, India.; ^2^ School of Science, Woxsen University, Hyderabad, Telangana 502345, India.; ^3^ Department of Computer Science and Engineering, BVRIT HYDERABAD College of Engineering for Women, Hyderabad, Telangana 500090, India.; ^4^ NanoBioNTech Laboratory, Department of Environmental Engineering, Florida Polytechnic University, Lakeland, FL 33805, USA.

## Abstract

Progressive neurodegenerative disease known as Parkinson’s disease (PD) is characterized by both motor and nonmotor symptoms that severely reduce the quality of life. Recent developments in soft robotics provide customizable, cozy, and less intrusive assistive devices, which provide promising answers to these problems. To develop an enhanced support system for people with PD, this article explores the potential of next-generation soft robotics, specifically hydrogel materials, integrated with artificial intelligence (AI) and augmented reality (AR) to provide an innovative solution for PD management. The integration of an AI copilot allows for remote monitoring and real-time adjustments, ensuring optimal performance and personalized care. The use of AR enhances human–computer interactions, offering an intuitive and immersive experience for both patients and healthcare providers. By leveraging these advanced technologies, our approach aims to substantially improve motor function, reduce symptoms, and enhance the overall quality of life for PD patients. This review outlines the key components, benefits, and potential impact of this novel approach, highlighting the transformative potential of combining wearable robotics, AI, and AR in the treatment of PD. The potential for creating novel healthcare solutions by combining soft robotics, functional materials, the Internet-of-Things (IoT), and machine learning (ML) is highlighted by this multidisciplinary approach.

## Introduction

Parkinson’s disease (PD) is a chronic neurodegenerative condition that affects motor function due to the death of dopamine (DA)-producing neurons in the substantia nigra. The disease worsens over time due to nerve cell degeneration in the basal ganglia, responsible for movement control. Movement problems arise from reduced DA synthesis caused by damaged or dead neurons [1,2]. Early symptoms are minor and appear gradually, such as tremors, difficulty getting out of a chair, soft speech, and slow writing. PD patients may not detect changes until they are noticed by friends or family. They often have a Parkinsonian gait, leaning forward, walking quickly in small steps, and reducing arm swinging. As the condition progresses, it affects both sides, with symptoms on one side being more severe than on the other [3,4]. PD has no known treatment, but some symptoms can be alleviated through drugs, surgery, and other therapies [5]. For patients not responding well to medication, deep brain stimulation may be beneficial [6]. During a surgical procedure, an electrical device and electrodes are implanted in the chest, stimulating specific brain areas controlling movement. Other therapies include physical therapy to improve balance, coordination, and flexibility. However, abruptly stopping the drug may have serious side effects. Traditional, inflexible robots have limitations in medical applications, particularly for patients with PD. Their rigid construction makes them heavy and potentially dangerous, as they may not conform to the human body’s sensitivity and dynamic nature. Additionally, rigid robots may not always meet the complex, erratic motions and diverse requirements of individuals with PD, which may require more precise mechanical control and precision [7].

**Fig. 1.**
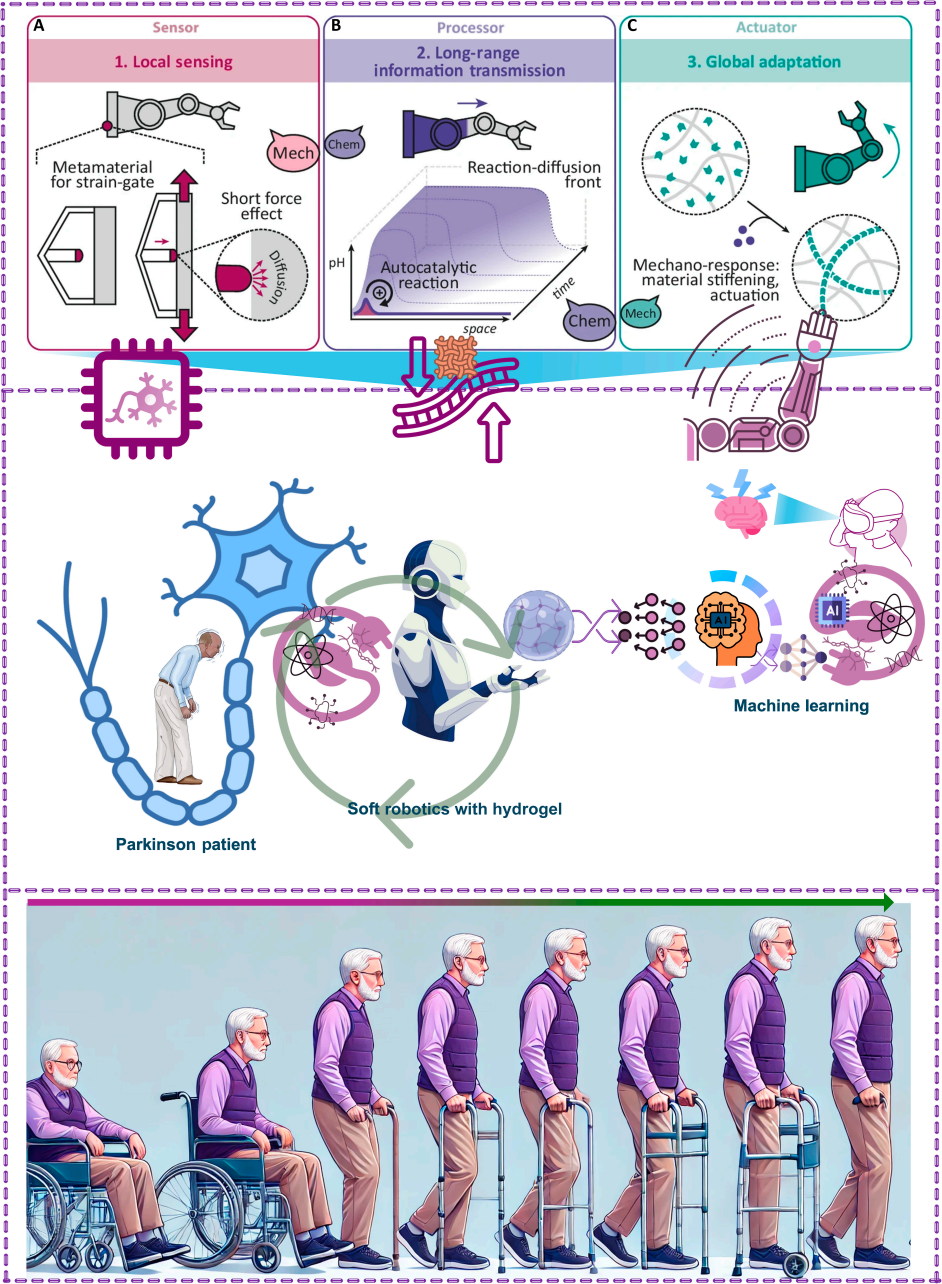
An integrated framework for advanced bioinspired soft robotics in Parkinson’s patient care. (A) Local sensing via mechanical metamaterials. (B) Long-range information transmission using chemical reaction–diffusion processes. (C) Global adaptation through mechanochemical actuation. The system synergizes hydrogel-based robotics, ML, and adaptive biomaterials to improve patient mobility and independence. Reproduced with permission [8]. Copyright 2024, *Nature Communications*.

The sensor–processor–actuator paradigm is used in chemomechanical and local-to-global information processing to create adaptable hydrogel muscles. A local mechanical trigger using gentle contact and short-range diffusive transfer is shown in Fig. 1A. Design of a metamaterial for strain-gated mechano-activation. Figure 1B illustrates the use of an autocatalytic reaction–diffusion front for chemical signal amplification and spatiotemporal transmission. System-level structural adaptability and mechanical reprogramming brought on by a downstream actuation are depicted in Fig. 1C. One example is the creation of supramolecular polymers. The speech balloons demonstrate how mechanical input is converted into a transportable chemical signal, which is then converted back into a mechanical output [8].

The world’s most creative and effective closed-loop controls are found in living things. Their behaviors are the result of several biofunctional modules working together logically and well-fashioned, rather than being produced by a single organ. Their seamless integration of muscles (actuation), skin (sensing), neural networks (computation), and brain (computation) gives them inherent qualities like robustness, flexibility, self-healing, and adaptability that are unmatched by traditional robots. With the help of new technologies (such as additive printing), a lot of studies on bioinspired soft robots have been done [9]. Soft robotics, characterized by its elasticity and flexibility, is becoming increasingly popular in the biomedical and healthcare industries for support and rehabilitation [10]. These robots are lightweight, affordable, and easy to assemble due to their flexible rubber-like elastomers [11]. They can change their stiffness and shape continuously, enabling them to perform specific tasks in harsh environments [12]. Soft robotics is becoming increasingly common in the biomedical and healthcare industries, particularly for PD patients. Material science plays a crucial role in the development of soft robotics, which uses functional materials like hydrogels, electroactive polymers, and shape-memory alloys to provide assistive and therapeutic devices [13]. Shape-memory alloys can deform and return to their original shape when heated, making them ideal for simulating human muscle motions. Electroactive polymers are responsive and adaptable, allowing for quick and easy actuation. These materials enable the design of exoskeletons and soft robotic gloves, which can help with daily activities and increase mobility. They also enable the design of sophisticated sensory feedback systems, allowing devices to respond in real time to human actions and requirements [14,15].

The Internet of Things (IoT) is a platform that can collect real-time motion measurements in PD patients by embedding wearable sensors in their clothes and connecting them to a medical database via mobile devices. These sensors record disease characteristics like gait patterns, tremor episodes, activity levels, and freezing of gait (FoG) [16]. The scientific and medical communities value data-to-knowledge translation to understand the gait differences between PD patients and healthy individuals. Researchers use gait segmentation to segment similar subsequences from the entire recording length, calculate spatiotemporal gait metrics, and analyze them using machine learning (ML) techniques to distinguish between normal and abnormal gait [17]. Soft robotic systems can provide intelligent, adaptive support, ensuring prompt and customized responses to varying PD symptoms [18]. Augmented reality (AR) technology is used to create an immersive and interactive interface for both patients and healthcare providers. Through AR devices, users can receive real-time feedback and guidance, enhancing their ability to interact with the system and perform daily activities more effectively.

The study aims to develop automated solutions for remote management and treatment of PD using wearable and portable technology. ML models are used to anticipate symptoms and make proactive adjustments to therapies, providing customized and dynamic treatment regimens. The research focuses on using cutting-edge materials like hydrogels for flexibility, comfort, and durability. The integration of these technologies into wearable robots is expected to standardize and optimize these robots, ensuring high-performance improvements in motor and sensory functions. The research aims to help patients with tasks, monitor motor symptoms, provide physical therapy exercises, and improve the overall quality of life by increasing independence and easing caregiver burdens.

## Functional Materials Used in Soft Robotics

**Fig. 2.**
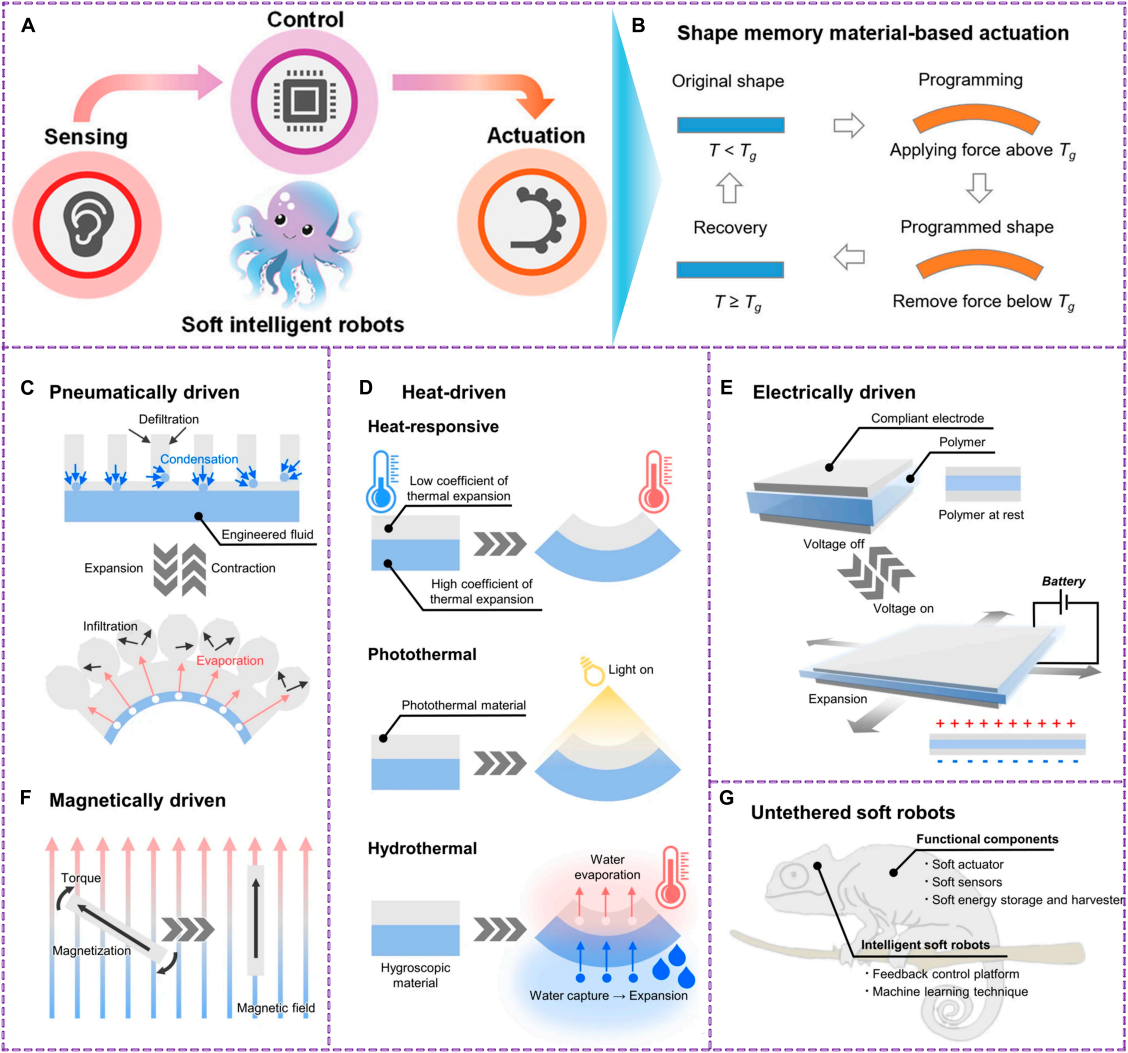
(A) Illustration of the functional components of soft intelligent robots, highlighting the integration of sensing, control, and actuation inspired by biological systems like an octopus. (B) Material-based actuation based on shape memory. When the temperature surpasses a specific threshold for the shape-memory effect, actuators are activated in shape-memory materials. Reproduced with permission [23]. Copyright 2023, *ACS Nano*. (C to G) Graphical representation of different types of untethered soft actuators and their actuating mechanisms for soft robotics. Reproduced with permission [24]. Copyright 2024, *Nature Communications*.

Soft robots, which simulate the continuous motions of animals and plants, are made of naturally compliant materials, drawing inspiration from real species [19]. Traditional hinges and bolts in soft robots are replaced by elastomers in actuators that change shape in response to inputs like pneumatic inflation. Rapid prototyping techniques make assembly easier, integrating morphing information into the design. However, these production techniques limit scalability, design flexibility, and robustness [20]. Depending on the material architectures and techniques used to manage magnetization profiles, intelligent magnetic soft robots can modify their structure in programmable and versatile ways [21]. Soft robotics is challenging to fabricate due to its laborious and time-consuming shape-morphing characteristic. Various synthesis methods, such as solvent casting, lithography, roll-to-roll technology, laser heating, spraying with spin technique, magnetized module assembly, and electron beam lithography, have been used to create soft robots. Figure 2A and B presents a future soft robot inspired by octopus-inspired robots, showcasing various soft robotic technologies. It outlines methods for achieving circuitry and actuation, including geometry-enabled, fluidic, and biohybrid strategies. Examples include serpentine or wavy copper wiring, deterministic design of auxetic metamaterials, microfluidic sensing, pneumatic actuation, biohybrid sensing, and muscle cell contraction under applied voltage [22,23]. Magnetically driven actuators use magnetic fields, pneumatically driven actuators measure volume change, and heat-responsive, photothermal, and hydrothermal types convert energy. Electrically driven actuators experience mechanical deformation due to electric field charge polarization (Fig. 2C to G) [24]. The main types of materials utilized in soft robotics are shape-memory polymers (SMPs) and elastomers. High-elasticity polymers, or elastomers, may stretch and deform greatly when subjected to stress before returning to their original shape [25]. They are frequently utilized in actuators, sensors, and soft grippers [26]. Thin soft robots, or TS-Robots, can move in several directions in the solid and liquid domains and can even switch between them to investigate hard-to-reach human-made settings, consisting of a thin soft dielectric elastomer actuator (TS-DEA; thickness, 1.2 mm) and many electrostatic adhesive pads (EA-Pads) constructed in symmetric and asymmetric configurations [27].

### SMP—Hydrogel

SMPs have the unusual capacity to revert to a predetermined shape in response to a particular stimulus, such as a change in temperature, pH, or light, making SMPs ideal for complex actuation devices [28,29]. Among all SMPs and composites, multifunctional hydrogels play a particularly useful role in the creation of cutting-edge smart structures [30]. Water-swollen polymer networks—hydrogels—are perfect for medical and bioinspired applications because of their exceptional biocompatibility and environmental stimulus reactivity [31]. Over the last 4 to 5 years, hydrogel-based microscale drug delivery carriers, including magnetic particles (MPs), have been investigated in the field of soft microrobotics [32]. Hydrogels offer unique benefits such as reversible reactions, adjustable magnetic responsiveness, simplicity of operation, biosafety, self-adaptability, intelligence, and compatibility with integration and shrinking. Leptocephali and similar species possess translucent hydrogel organs and tissues, enabling quick movement and blending in with their surroundings [19].

Recent research has shown that hydrogel systems can combine chemical activation and electrical sensing through stiff integrated circuits with wavy interconnects, enhancing bonding between hydrogels and various materials, thereby improving their integration [22]. Hydrogels may expand or decrease in bulk isotopically while absorbing or losing water in the networks, making them more useful for aquatic mobility [33–35]. A soft robot with an octopus-inspired hydrogel adhesive (OHA) can easily adhere to biological tissues and disengage. The hydrogel’s dome-like protuberance structure allows for easy detachment by temperature changes and strong tissue attachment underwater. This tiny, soft OHA robot performs biomedical tasks effectively within the body [36]. Particularly, hydrous carbon black added to hydrogels increases the soft sensor’s sensitivity [37].

**Fig. 3.**
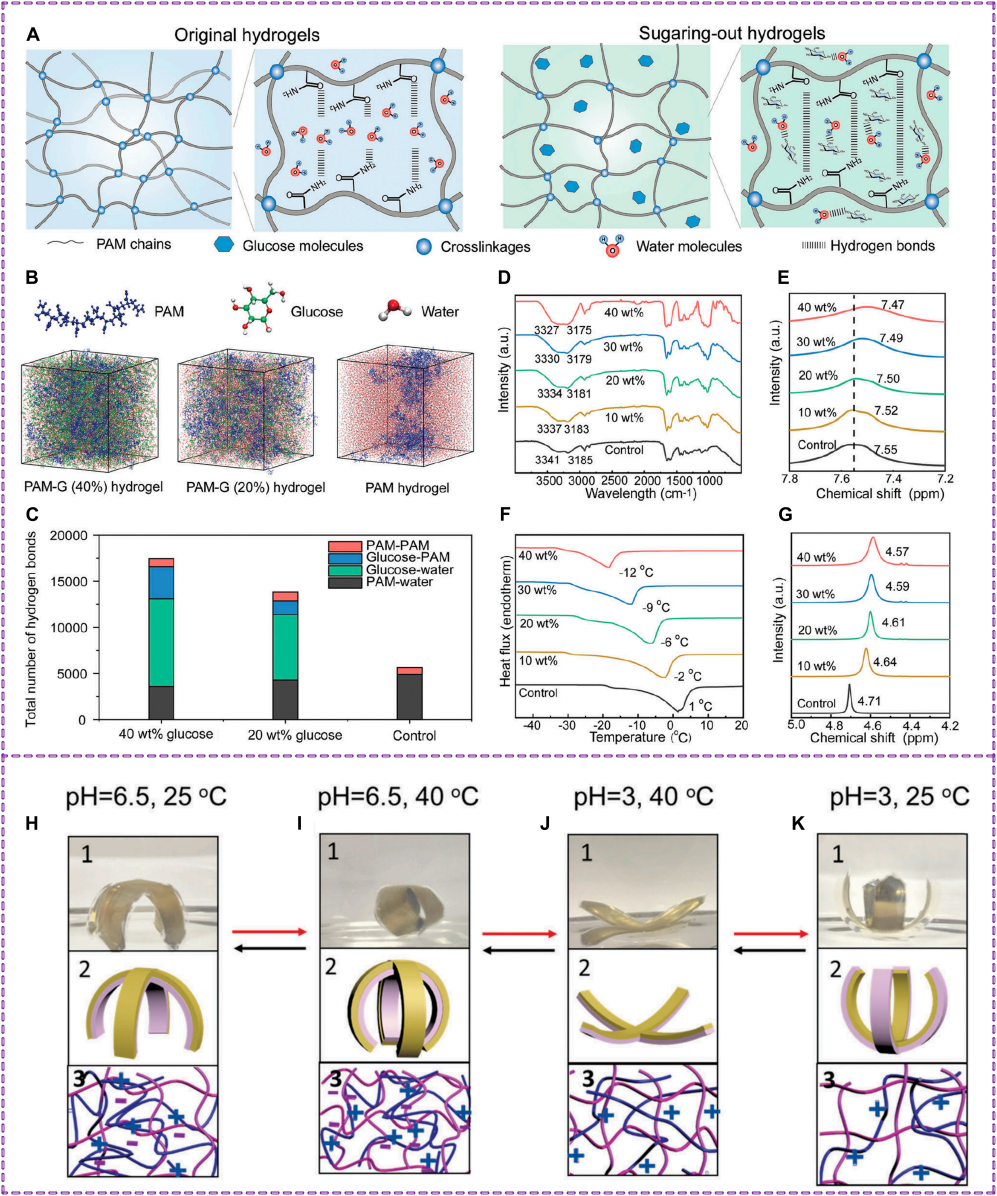
(A) Schematic representation of the material design strategy. (B) Snapshots from molecular dynamic simulation displaying 3 distinct hydrogel compositions. (C) Number of hydrogen bonds determined by molecular dynamic simulation. (D) Fourier transform infrared (FTIR) spectrum of PAM and PAM-G hydrogels with varying glucose contents. (E) Magnified ^1^H nuclear magnetic resonance (NMR) spectrum of PAM and PAM-G hydrogels with varying glucose contents. (F) Differential scanning calorimetry (DSC) curves of PAM and PAM-G hydrogels with varying glucose contents. (G) ^1^H NMR spectrum of water/glucose solution with varying glucose contents. Reproduced with permission [38]. Copyright 2024, *Advanced Functional Materials*. (H to K) Bent bilayer structures: (1) photograph, (2) schematic diagram, and (3) proposed arrangement of polymer chains at the indicated solution temperature and pH. Reproduced with permission [39]. Copyright 2017, *Journal of Materials Chemistry B.*

Hydrogels’ high free water content limits their mechanical performance and environmental resilience. Polyacrylamide (PAM) hydrogels are toughened using glucose, facilitating hydrogen bonding and interchain interactions [38]. This high hygroscopicity makes the hydrogels resilient to extreme conditions. PAM-G hydrogels are used as multimodal sensors in soft robotics and opto-mechanical sensors, with no negative health or environmental effects. In addition to (Fig. 3A to G). Li et al. [39] created semi-IPN (interpenetrating polymer network) hydrogel-based bilayer actuators by generating a hydrogel based on poly(N-isopropylacrylamide) (pNIPAm) in the presence of poly(diallyldimethylammonium chloride) (pDADMAC) on a gold-coated polydimethylsiloxane (PDMS) layer. These bilayers displayed unique bidirectional bending behavior in response to temperature and pH changes, a behavior distinct from that of pNIPAm-based bilayers. The bending direction and degree could be adjusted by tuning the hydrogel layer composition shown in Fig. 3H to K. The bilayers can be used as stimulus-induced grippers and controlled small-molecule delivery, making them useful for biomedical applications.

### 2D, 3D, and 4D material-integrated hydrogel

Soft robots can perform tasks in extreme heat and environmental hazards, maintaining comfortable temperatures because of 2-dimensional (2D) material-integrated hydrogel (2DM/H) skins [40,41]. One of the best strategies for promoting wireless soft drives is photoresponsive soft robotics because of their exceptional photoresponse qualities, ductility, and good biocompatibility [42]. These hydrogels may acquire photodeformation and carry out a wide range of intricate actions, including bending, turning, stretching, and crawling. Huang et al. [43] formed various conversions and cross-linking densities over polyhydrogel by dynamically and spatially controlling light using a digital projector. The printed hydrogel’s mechanical properties and uneven swelling ratio enable it to expand into 3D forms, a technique used in soft actuation. Studies on programmable multi-morphing 3D topologies with composite hydrogel sheets show potential for soft robotic bulk [44]. Furthermore, the 3D-printed electroactive hydrogel (EAH) model provides controlled soft robotic manipulation and mobility under the circumstances of reacting to an electric field [45,46]. Composite hydrogel architecture with localized components can achieve anisotropic swelling behavior, enabling a revolutionary technique for biomimetic soft robotic 4D printing, including the creation of cellulose fibrils [47,48]. Gladman et al. [49] created anisotropically swelling localized composite hydrogels by aligning cellulose fibrils along predetermined 4D printing paths. One of the most creative and promising methods to create highly functionalized soft actuators is to include MPs in 3D-printed smart material [50–52].

Magneto-active hydrogels (MAHs) have been the subject of in-depth investigation as they are thought to be the answer to creating increasingly intricate components with superior biodegradability and crack-healing capabilities [53]. Researchers have proposed transformable hydrogel composites for use in gradient hydrogel-based robots, bilayer hybrid soft robotics, various actuators, and universal robotic “skins” through 3D/4D printing, layer-by-layer polymerization, photolithography, and mask printing [54]. Composites can be manipulated using various stimuli, enabling the creation of more effective and comfortable tremor-suppressing tools through the combination of 3D/4D printing, smart material science, and modeling and embodiment control characteristics [55]. Using direct-ink-write (DIW) printing, Cheng et al. [56] created 3D freeform structures of chemically and physically cross-linked hydrogels by utilizing biocompatible alginate as a rheological modifier. The easy, precise, and consistent manufacture of 4D-printed hydrogels has drawn a lot of interest lately [57]. In this context, the hydrogels polydopamine (PDA), poly(3,4-ethylenedioxythiophene):polystyrene sulfonate (PEDOT:PSS), and polyacrylamide (PAAM) are frequently employed to achieve toughness and biocompatibility and to verify the hydrogels’ suitability for 3D printing into unique structures [58,59]. Polyacrylamide nanocomposite hydrogels, created by polymerizing acrylamide with PDA-modified carbon nanotubes, are biocompatible, self-healing, self-adhesive, electrically conductive, and flexible strain or pressure sensors, making wearable sensor applications feasible [60,61]. The sensitivity of a hydrogel as a strain sensor is indicated by its gauge factor (GF). A higher GF indicates greater sensitivity to strain, which is ideal for soft robotic sensors.

The study by Jung et al. [62] presented a multi-deformable double-sided (MDD) DA-sensing probe that integrates working, reference, and counter electrodes on a single probe. The probe achieves high DA sensitivity and selectivity through enzyme immobilization on 3D nanostructures. The serpentine design ensures high stretchability and manages stress. The probe has been successfully implanted into the brain for real-time in vivo monitoring of DA levels in rodents and hemi-PD mice. This deformable probe has potential for neurodegenerative disease study and treatment.

### Conductive hydrogels

Even conductive hydrogels made of natural biopolymers have gained attention as potential components for stretchy and wearable sensing systems [63–65]. These hydrogels provide enhanced mechanical characteristics and biocompatibility by using nontoxic and renewable biopolymers such as gelatin, chitosan, silk fibroin, and cellulose [66]. It is regarded as one of the finest options for next-generation flexible wearable sensors. Ions could be dissolved into water to create hydrogels, which were incredibly elastic and soft like tissue ionic conductors [67,68]. The composite conductive hydrogel, a flexible wearable sensor, was enhanced in conductivity and sensitivity by combining ultralong silver nanowires and modified carbon black nanoparticles [69]. A flexible wearable sensor made of a composite hydrogel combining polyvinyl alcohol (PVA), tannic acid (TA), and polyacrylamide (PAM), integrated with silver nanowires (AgNWs) and hydroxylated carbon black (CB–OH) with conductive material has been developed, enhancing its conductivity and making it suitable for full-range human motion detection, including activities like talking, walking, jumping, and bending [70]. In a work inspired by chameleon skin, Bai et al. [71] introduced dual-sensing ironic skin (DSI-skin), which is constructed of electromechanical and mechanochromic material with various sensing functions, antibacterial characteristics, and an antifreeze energy source. Ionic conductivity was imparted to the hydrogel by adding Al^3+^ ions. The cable-driven continuum robot (CDCR) is a lightweight, safety-conscious soft robot that uses skin-like hydrogel sensors made of ionic conductive polyacrylamide/alginate/nanoclay polymeric composite hydrogels. These sensors are sensitive, steady, and reliable, and can be manually handled for portrait drawing [72].

Hydrogels offer biocompatibility, softness, responsiveness, and ease of fabrication, making them ideal for applications in soft robotics, human–robot interaction, medical rehabilitation, and other biomedical engineering and robotic fields. Table 1 compares the mechanical strength, biocompatibility indices, and responsiveness metrics to make the material selection rationale more evident, where material properties are crucial for functionality and integration with biological systems.​

**Table 1.** Key mechanical and functional properties of various hydrogels

Hydrogel type	Chemical composition	Electrical conductivity	Gauge factor	Tensile strength (kPa)	Elastic modulus (kPa)	Biocompatibility index	Stimuli responsiveness	Relevance to PD applications	Ref.
MXene-based	Alginate-based	High (~10–50 S/cm)	~30–100+	45	12	High (cell viability >90%)	pH-sensitive (pKa ~6.8)	Soft robotic actuators and real-time electrophysiological sensors	[147,148]
	Gelatin-methacrylate			60	20	Moderate (cell viability ~80%)	Thermo-responsive (Tg ~32 °C)		[149]
Carbon nanotube (CNT) based	Polyacrylamide	High (~1–10 S/cm)	~20–80	75	35	High (low cytotoxicity)	Dependent on purity	High-sensitivity strain sensors in soft robotics	[150,151]
Reduced graphene oxide	Polyvinyl alcohol	Low (~10^−6^–10^−4^ S/cm)	~0.5–1	0.42 MPa	75	High	Humidity, temperature	Flexible sensors for wearable monitoring	[152,153]
Double-network	Polyacrylamide + alginate/chitosan	Moderate (~10^−3^ S/cm)	~1–5	62 kPa	100–1,000	Excellent	Ionic strength, temperature	Robust actuators, long-term wearable supports	[154–157]

## Design of Soft Robotic Systems for PD

**Fig. 4.**
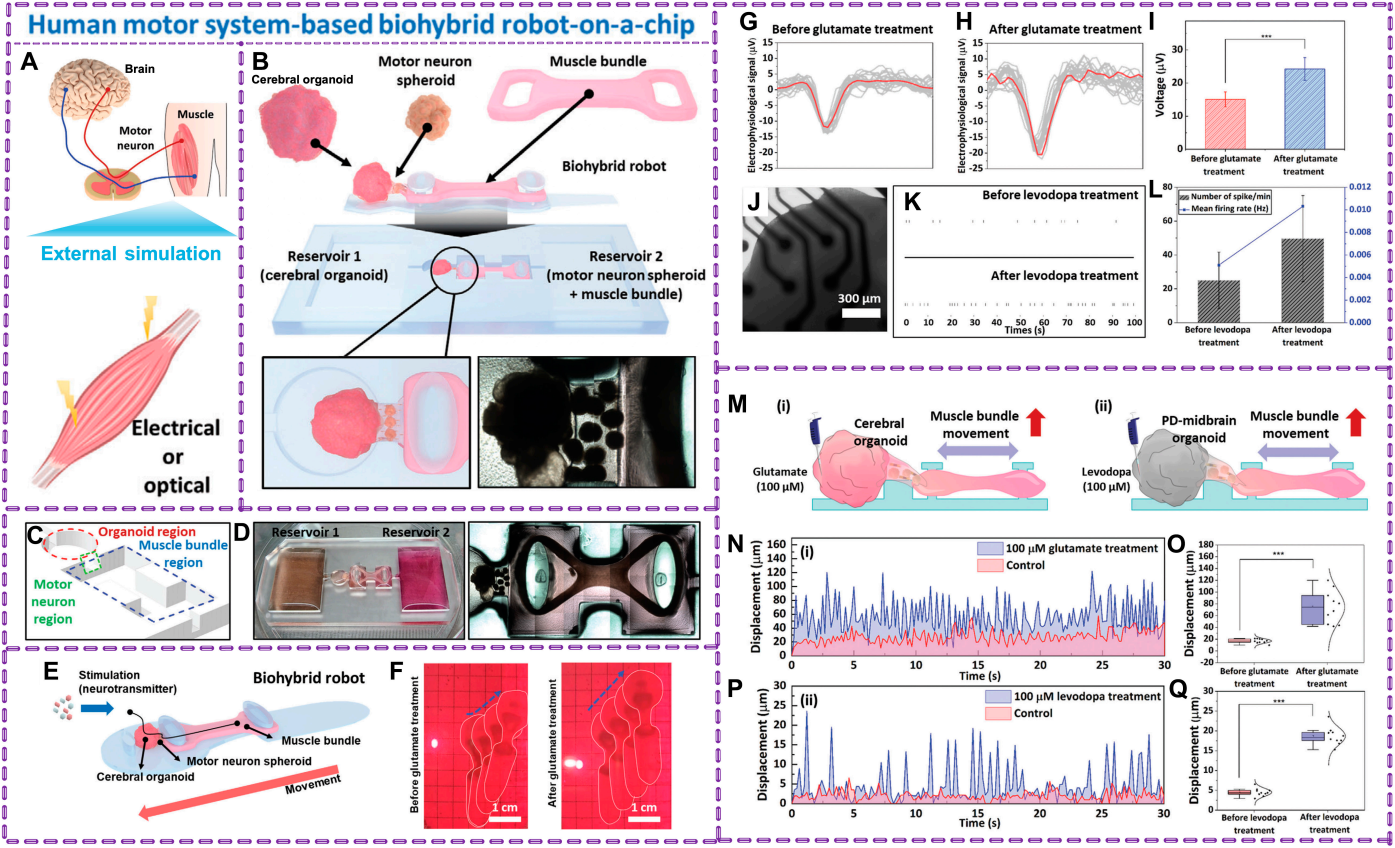
(A) Diagrammatic representations of the human motor system and spontaneous and externally stimulated muscle activity. (B) Schematic illustration of the biohybrid robot-on-a-chip based on the human motor system, which is made up of the muscle bundle, motor neuron spheroid, and brain organoid on a solid substrate. (C) Diagrammatic illustration of the muscular bundle (blue), motor neuron (green), and organoid (red) areas. (D) Optical representation of a biohybrid robot-on-a-chip based on a human motor system. (E) Biohybrid robot’s schematic picture. (F) Comparison of the movement of a biohybrid robot before and after glutamate therapy. Brain organoid electrophysiological signal analysis (G and H) before and following 100 × 10^−6^ m glutamate therapy. (I) The brain organoid’s electrophysiological signals are quantified. ***P < 0.001. The SD from 19 measurements is shown by the error bars. (J) The microelectrode array (MEA) system’s optical representation of the PD-midbrain organoid. (K) PD-midbrain organoid spike raster plot before and after 100 × 10^−6^ m levodopa therapy. (L) PD-midbrain organoids’ mean firing rate and number of spikes before and after levodopa therapy. (M) Diagrams showing the movement of the muscle bundles in (i) a biohybrid robot-on-a-chip based on a cerebral organoid and (ii) a biohybrid robot-on-a-chip based on a PD-midbrain organoid. (N and O) Muscle bundle movement produced by glutamate in the biohybrid robot-on-a-chip based on the cerebral organoid. (P and Q) Movement of muscle bundles triggered by levodopa in the biohybrid robot-on-a-chip PD-midbrain organoid. ***P < 0.001. The SD from 10 measurements is shown by the error bars. Reproduced with permission [75]. Copyright 2024, *Advanced Science.*

PD requires new therapies to delay progression and control motor symptoms in advanced stages. Neurotrophic factor-loaded hydrogels with stem cells are promising, potentially increasing DA content and promoting stem cell differentiation [73]. The soft robotic system aims to provide personalized, flexible, and timely support to Parkinson’s patients, using wearables, an integrated IoT framework, ML algorithms for adaptive control, and a user interface for immediate feedback and interaction [74]. Neuromuscular interfaces and flexible electronics enable embodiment feedback technologies to enhance agency and sensing of human intention. These technologies create a feedback loop by sending data about the external environment or the human body’s condition to the nervous system. Shin et al. [75] proposed a human motor system-based biohybrid robot-on-a-chip to evaluate the drug effect on neurodegenerative diseases (Fig. 4). The robot, composed of a brain organoid, motor neuron spheroids, and muscle bundle, was used to study PD. The patient-derived midbrain organoid was incorporated into the robot-on-a-chip, and the drug effect was measured by increasing muscle bundle movement in response to levodopa.

Sensors must be incorporated into soft robotic systems to provide both proprioceptive and exterior perception for efficient use, to more closely resemble biological systems [76]. Everyday actions need leg mobility, although many people suffer on a regular basis because of impairments to their leg motion. Soft robotics makes use of malleable, soft materials that might result in a robotic system that is less expensive.

### Wearables

Wearable technology, including clothing, watches, eyeglasses, and shoes, can help prevent tremors from medical treatments. However, bulky designs limit hand movement. Simulated tremor suppression is used to assess glove performance in both dorsal and palmar designs, ensuring better performance in preventing adverse effects [77]. A wearable exoskeleton called the soft wearable assistant for gait (SWAG) helps with hip flexion. Pneumatic rotary actuators (PRAs) are used by the device to transfer torque to the leg in the direction of hip flexion [78]. In PD, FoG is a severely disruptive gait disorder that results in an unintentional halt when walking. The soft robotic apparel facilitates limb progress during the walking swing phase by providing bilateral assistive hip flexion torques. There are not many effective therapies for FoG since their effects are mild and fleeting. By working with biological muscles, the wearable garment creates assistance moments through the use of cable-driven actuators and sensors [79,80]. The functional apparel worn around the waist and thighs serves as anchor points and interfaces for flexible cable-driven actuators and sensors, measuring applied force and wearer movement. In addition to traveling 49 ± 11 m (+55%) further than when walking alone, walking quicker (+0.18 m s^−1^), and improving gait quality (−25% in gait variability), FoG was instantly abolished during interior walking with help (0% versus 39 ± 16% time spent freezing while walking unassisted).

**Fig. 5.**
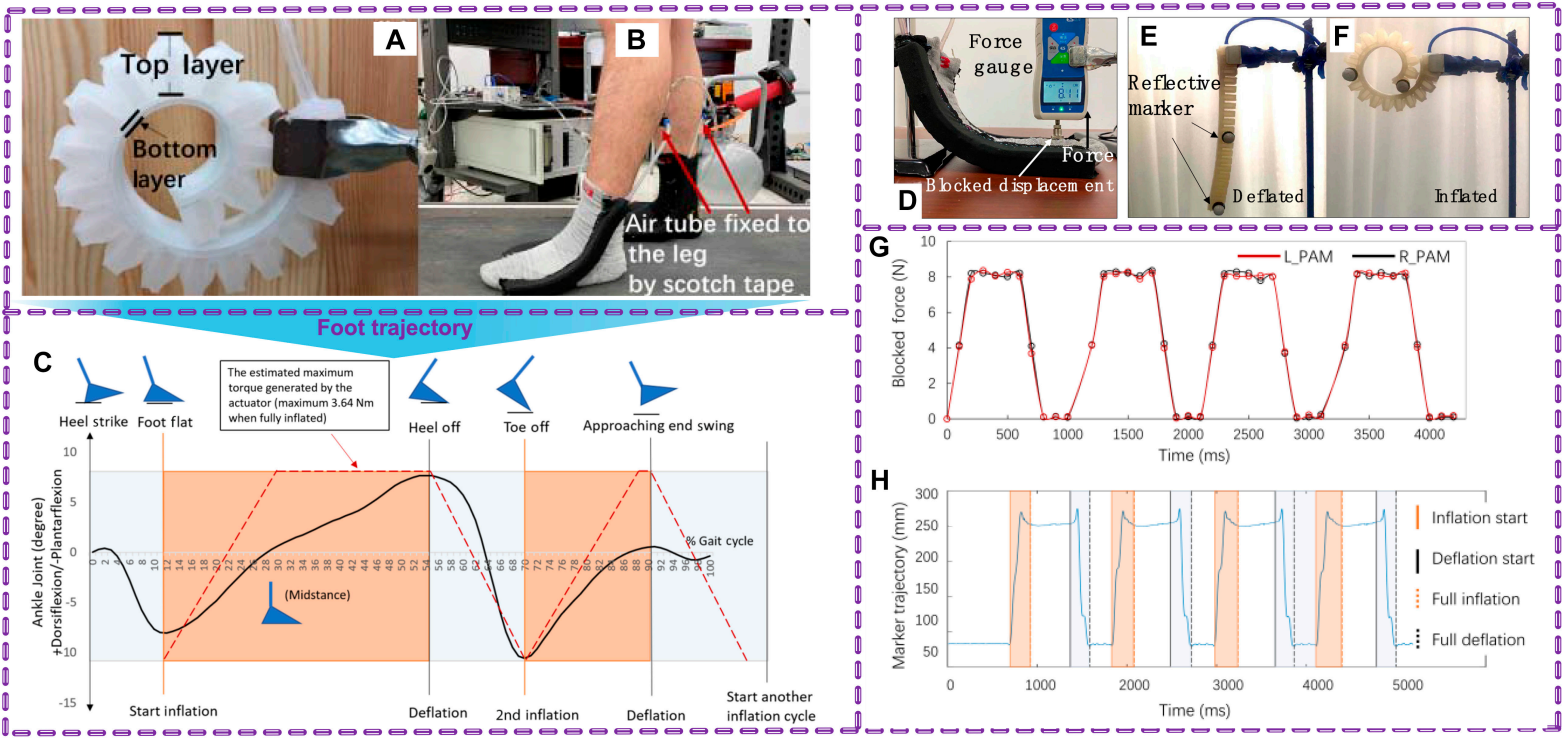
The design of the PAM. (A) Inflated PAM. (B) Actuators worn around the ankle. (C) Foot trajectory across a gait cycle with actuator pressure phases (inflation/deflation) aligned to gait events such as heel-strike and toe-off. (D) Blocked displacement characterization test of the PAM. (E) Test for the deflation speed and (F) inflation speed. (G) Resulting blocked force of the PAM (showing 4 inflation/deflation cycles). L_PAM, PAM on the left side; R_PAM, PAM on the right side. (H) Vertical trajectory of the marker at the far end of the PAM (during the 4 inflation/deflation cycles). Reproduced with permission [81]. Copyright 2021, *IEEE Xplore.*

A study has proposed a wearable soft robotic intervention to improve gait variability in older adults [81]. The system uses customized pneumatic artificial muscles to assist ankle dorsiflexion during walking. Twelve participants with low fall risks and 12 with medium-high fall risks participated in the experiment. Results showed that the intervention reduced step length variability for elderly people with medium-high fall risks, suggesting that it could be an effective fall prevention solution for older adults (Fig. 5).

**Fig. 6.**
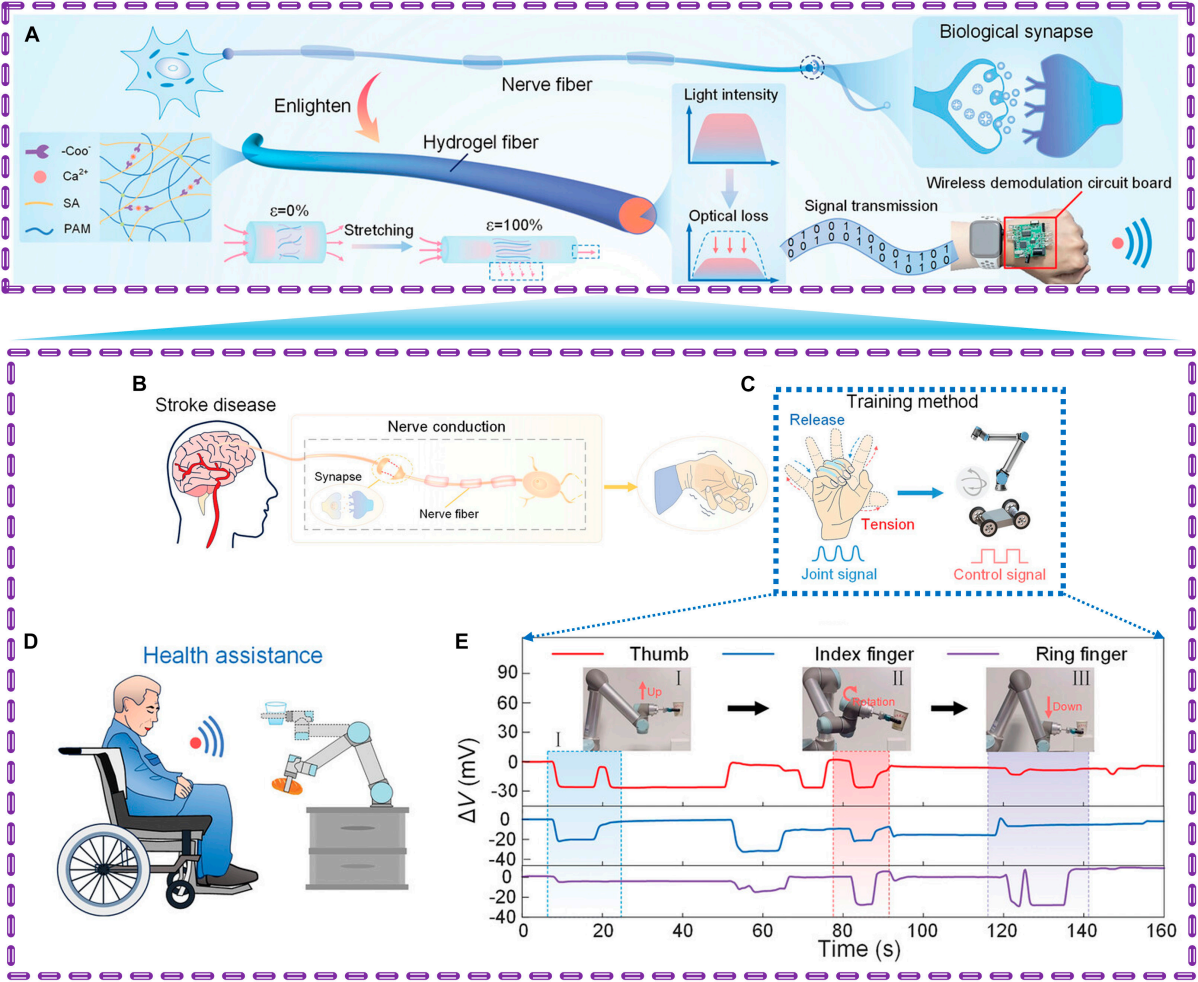
(A) Design, function, and application of the HOWS sensor—a mechanical device-based finger dexterity rehabilitation activity. (B) Comparison of stroke patients’ rehabilitation exercises for their finger joints before and after. (C) Design of 2 rehabilitation techniques for training finger joint rehabilitation. (D) Robotic arm control-based finger control experiment. (E) Signals are sent when the robotic arm moves. Reproduced with permission [86]. Copyright 2024, *Advanced Science.*

Since insoles come into close contact with the foot, shoes with smart insoles are mainly utilized for recording gait metrics. Numerous sensors included in smart shoes allow them to track a variety of metrics, including toe-off, heel strikes, step counts, and foot pressure [82]. The inertial measurement unit (IMU) and pressure sensors are used in the study’s smart insole-based approach to continuously detect foot-shuffling steps and estimate the length of time spent shuffling throughout a step cycle [83]. A wearable exoskeleton soft rehabilitation glove is recommended for patients with dementia who have lost hand function, have limited range of motion, and have insufficient finger muscular power for passive or auxiliary rehabilitation exercise training [84]. The goal of Wirekoh et al. [85] was to create a new type of lightweight, compact bending actuator that, when integrated into a wearable assistive technology called fiber-reinforced bending pneumatic artificial muscle (BPAM), could actively control hand tremors. The ability of the fiber-reinforced BPAM to function within the dynamic regime of hand tremors indicates its potential for further development into a device capable of actively controlling hand tremors. Later, Li et al. [86] proposed a hydrogel-based optical waveguide stretchable (HOWS) sensor with a double network structure for high stretchability and sensitivity (Fig. 6). The sensor’s flexible, smart bionic fabric enhances comfort. A circuit board for wireless signal transmission improves portability. A speech recognition and human–machine interaction system is built, and a convolutional neural network (CNN) algorithm is integrated for disease assessment. This smart tele-healthcare system has the potential for early warning and rehabilitation monitoring of neurological diseases.

**Fig. 7.**
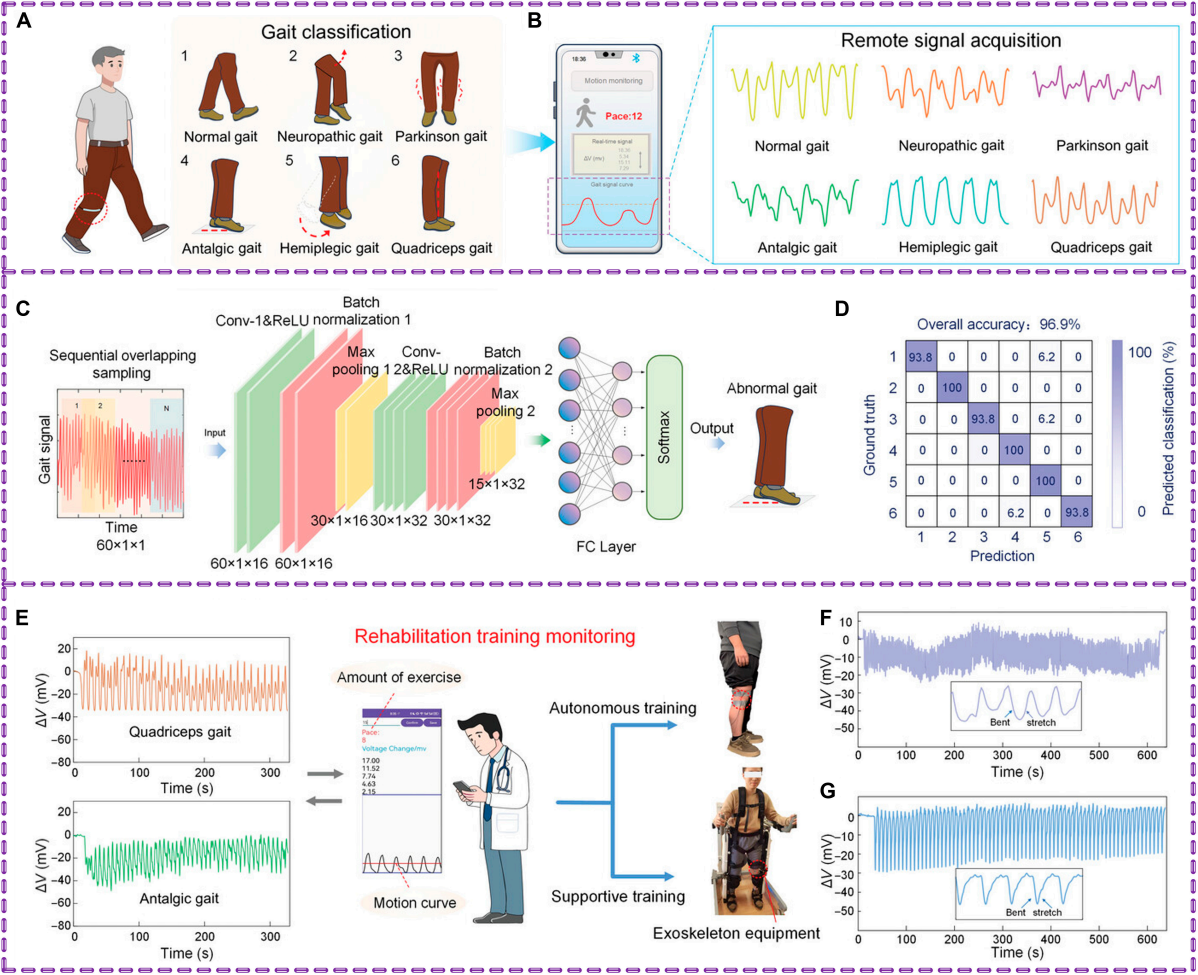
Applications for remote monitoring and the identification of gestures and gait. (A) Distinguishing aberrant gait characteristics. (B) Using a mobile application to record abnormal gait signal characteristics. (C) The 2D convolution method model is used to classify and analyze gait signals. (D) Classification and recognition confusion matrix of gait signals. (E) The HOWS sensor is combined with a smartphone app for autonomous monitoring and aided walking exercises. (F) Signal alterations during self-directed activity. (G) Signal alterations while exercising using a machine. Reproduced with permission [86]. Copyright 2024, *Advanced Science.*

Smartwatches are a crucial component of smart consumer wearable technology, recording various physiological metrics like heart rate, blood sugar level, steps taken, calories burned, and sleep quality. They also use motion sensors like gyroscopes and accelerometers to gather data on arm movement [87]. Wearable technology must be comfortable to use for extended periods, which is why hydrogels are important for patient compliance [12,88]. Qin and colleagues [89] reported on a hydrogel strain sensor that demonstrated strong tensile strength (166 kPa), super-tensile properties (>1,600%), and low latency in the detection of both moderate physiological activity and robust human activity. This hydrogel forms a PAM network with high toughness and electrical conductivity that may find utility in wearable medical electronics. PVA–pectin–pectin-tannic acid (PPT) hydrogels are a type of ionotropic hydrogel that can be printed in 3D and are self-adhesive, self-healing, and conductive. With a GF of 2.5, the hydrogels show good electrical conductivity, appropriate sensitivity, and a wide sensing range (about 2,000%) [90]. Wearable strain sensors for tracking different human movement behaviors may be created by directly printing the intricate self-healing pattern onto a flexible substrate [91]. Abnormal gait formation can be caused by central nervous system damage, muscle weakness, or lower limb pain. These conditions affect muscles, joint movements, and neural control during walking, leading to different gaits such as neuropathic, festinating, antalgic, hemiplegic, and quadriceps gaits. In this study, a HOWS sensor was attached to the knee joint to artificially simulate various abnormal gaits [86]. A CNN was used to classify and analyze the collected data. The results showed an accuracy of 96.9%, better than relevant algorithms, highlighting the potential of HOWS sensors in early disease detection. Monitoring movement trajectories via smartphones helps healthcare providers understand patients’ joint activities and develop targeted exercise plans. This technology could help healthcare providers better understand patients’ joint activities and develop more effective exercise plans (Fig. 7).

One of the most important clinical issues with PD is falling. Fall hazard ratios in people with PD may be measured in real life using body-worn sensors worn as a necklace [92]. The next generation of human-in-the-loop control aims to create user-friendly, natural control strategies for wearable robots, integrating multiple human responses to improve interaction and enhance comfort, despite differences in needs for exoskeletons and prostheses [93,94]. Prostheses are used for reconstruction, while exoskeletons enhance motor and sensory functions. Human-in-the-loop control integrates people into wearable robots, adjusting settings based on factors like muscle activity, synergy, metabolic cost, gait symmetry, user choice, or comfort. This method optimizes user reactions and reduces or optimizes their reactions [95]. A prototype system was developed by Seshadri [96], which uses granular jamming and soft robotics to detect and suppress tremors automatically. To identify whether a tremor is present, the gadget detects vibrations from hand motions, analyzes the frequency, and then activates a wrist cuff that is filled with coffee grounds.

Even while wearable PD solutions have demonstrated encouraging outcomes in controlled laboratory settings, their practical use frequently shows significant shortcomings. When used outside of clinical settings, devices that exhibit excellent accuracy and dependability in short-term trials may face problems with human variability, ambient noise, and normal wear and tear. Data quality is compromised by elements including uneven sensor placement, motion artifacts from everyday activities, and device deterioration with time. Furthermore, compared to supervised trials, user compliance in real-world settings is typically lower, especially if the devices are invasive, need regular maintenance, or disrupt daily routines. In addition to strong engineering solutions, iterative testing in a variety of uncontrolled settings, continuous user input, and support systems to encourage long-term adoption are also necessary to close this lab-to-life gap.

### IoT and ML integration

The soft robotic system for PD patients integrates state-of-the-art functional materials, ML, and IoT-enabled monitoring to provide comprehensive support and therapeutic [97,98]. When combined, each element offers unique benefits that significantly enhance patient care. The smooth integration of these technologies to provide reliable, astute, and approachable treatments is where this field’s future rests [97]. IoT and ML are critical for intelligent operation, but functional materials are necessary for the device’s mechanical components [99,100]. To develop efficient biomimetic robotic systems, the intersection of ML and electronic skins has been thoroughly researched [23]. Through linked sensors, IoT makes it possible to continuously monitor a patient’s state and provides useful data for both short- and long-term studies [101,102]. Meanwhile, large datasets gathered from the bioelectronics industry have shown that ML is effective. Numerous opportunities in previously unheard-of diagnostics, therapies, and other fields have been made possible by the combination of ML with hydrogel-based soft bioelectronics [103].

**Fig. 8.**
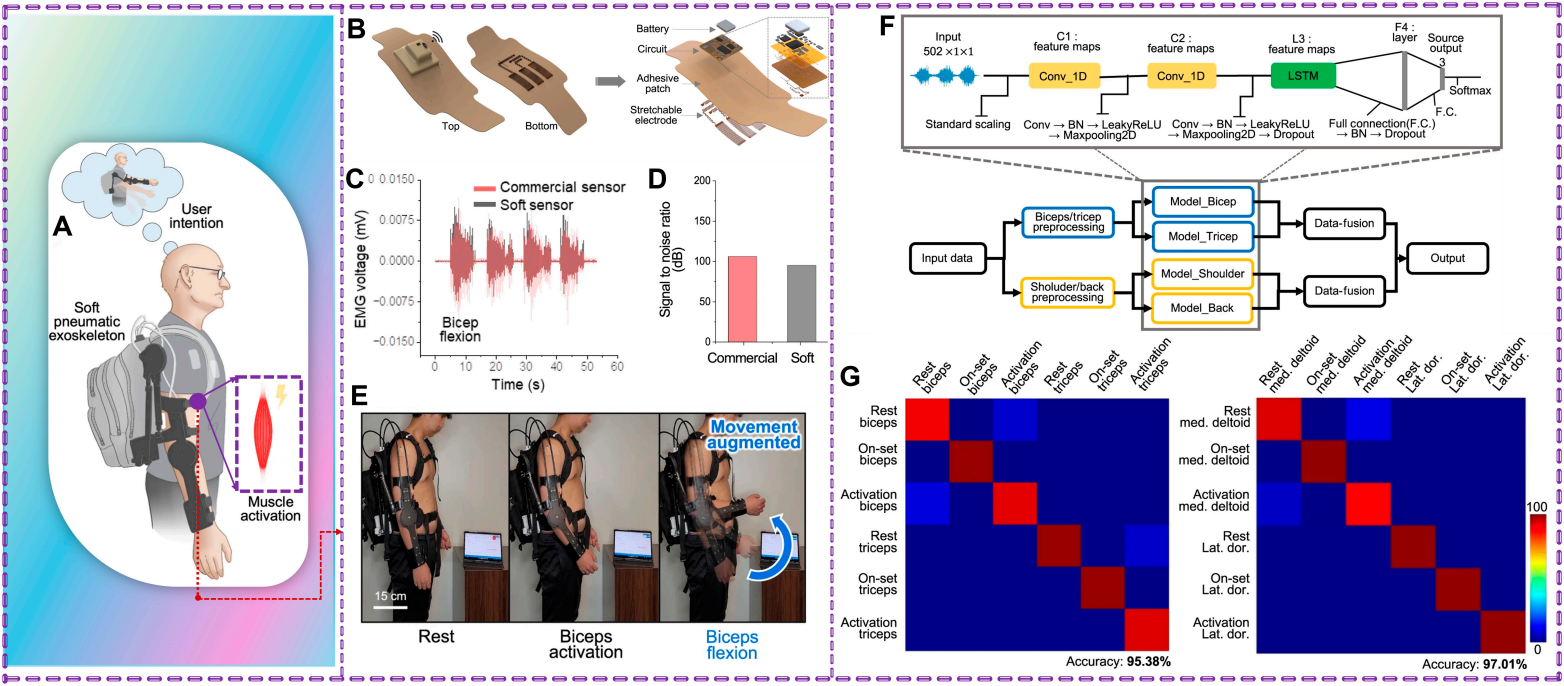
(A) Overview of the system architecture featuring an intelligent upper-limb exoskeleton with embedded soft sensors and soft actuators. (B) Graphical illustration of the soft wireless sensor package (left) and its exploded view (right), showing the stretchable electrode, adhesive patch, battery, and flexible circuits. (C) Comparison of electromyography (EMG) signals from the soft sensor and a commercial sensor during intermittent elbow flexion. (D) Signal-to-noise ratio of the EMG data measured in (C), showing similar measurement quality. (E) Demonstration of augmented motions in real time in sequential order, which includes elbow flexion and elbow extension. (F) Model architecture of the cloud-based deep learning algorithm. (G) Confusion matrices showing the model classification accuracy of 95.38% and 97.01% for biceps/triceps (left) and medial deltoid/latissimus dorsi (right), respectively. Reproduced with permission [106]. Copyright 2024, *npj Flex Electron.*

Wearable sensors enable continuous monitoring, while microcontrollers and edge devices handle local data processing. Communication modules ensure smooth connectivity, while cloud platforms enable enhanced data analysis and remote monitoring [104]. Shu et al. [105] demonstrated a shape-sensing electronic skin (SSES) based on a differential piezoelectric matrix that can identify surface conformations with little interference from stretching, pushing, or other external stimuli. As a proprioceptive sense, it is then included in soft robots to help them regain their form while moving. Lee et al. [106] developed an intelligent upper-limb exoskeleton system that uses deep learning to predict human intention for strength augmentation. The system uses embedded soft wearable sensors to collect real-time muscle activities and compute the user’s intended movement. The cloud-based deep learning predicts 4 upper-limb joint motions with an average accuracy of 96.2% (Fig. 8). The intent-driven exoskeleton reduces human muscle activities by 3.7 times on average compared to the unassisted exoskeleton.

A wearable smart anklet that is portable was created to measure the gait features of PD patients to classify and identify them by using feature engineering and an enhanced *K*-nearest neighbor (KNN) multi-feature classification method [107]. Intuitive interaction with the robotic system is made possible by natural language processing (NLP) and gesture recognition systems. Voice instructions or natural gestures can be used by patients to operate the gadgets [108,109]. For example, a reflective ink coating was applied to the finger surface to provide extensive tactile sense, and neural networks were successfully used to classify items based on their forms [110]. Through trial and error, people can acquire reinforcement learning, which remembers and rewards positive behavior to promote human–robot interaction [111]. A bimodal self-powered flexible sensor (BSFS) was developed using the magnetoelastic effect and a triboelectric nanogenerator. An anthropomorphic soft robotic hand with multimodal sensing skills was created by integrating BSFSs into soft fingers, using a CNN [112]. A vision-based deep learning framework called depth enhanced hand posture intention network (DEEPOSE-Net) is used to detect intentions regarding multiple hand postures and a soft robotic glove for stroke survivors and healthy individuals [113]. Based on the resistance response of conductive hydrogels, Shi et al.’s [114] work provides an E-skin with mixed electronic and ionic conductivity that may concurrently accomplish exteroceptive and proprioceptive. A new hydrogel sensing system, combining multiwalled carbon nanotubes (MWCNTs), sodium chloride (NaCl), and PVA/PAM additives, has been developed to allow state awareness in soft robotics. This innovative system can anticipate changes in attitude in soft robots resulting from various degrees of pressing, hot pressing, bending, twisting, and stretching.

Using a different strategy, Park et al. [115] developed biomimetic robotic skin using hydrogel elastomer hybrids. The sensor uses a hydrogel encased in silicone elastomer to mimic skin and human hand tissue. It detects even the smallest contact on the skin’s surface and calculates force using vibration-sensitive microphones. High-accuracy touch measurement is achieved by relaying signals to deep neural networks. Furthermore, Kim et al. [116] developed a unique synthetic hydrogel conductor that is incredibly elastic and self-healing by using all ocean biomaterials, such as diatom, chitosan, and catechol. The skin-attachable, self-powered tremor sensors and stretchy TENG that can gather energy from human motion and track the health of PD patients were made possible by the usage of the catechol–chitosan–diatom hydrogel (CCDHG) conductor [116]. Furthermore, the CCDHG-TENG and an M-shaped Kapton film were especially used to develop a self-powered tremor sensor, which was then applied to diagnose PD patients using an ML technique.

**Fig. 9.**
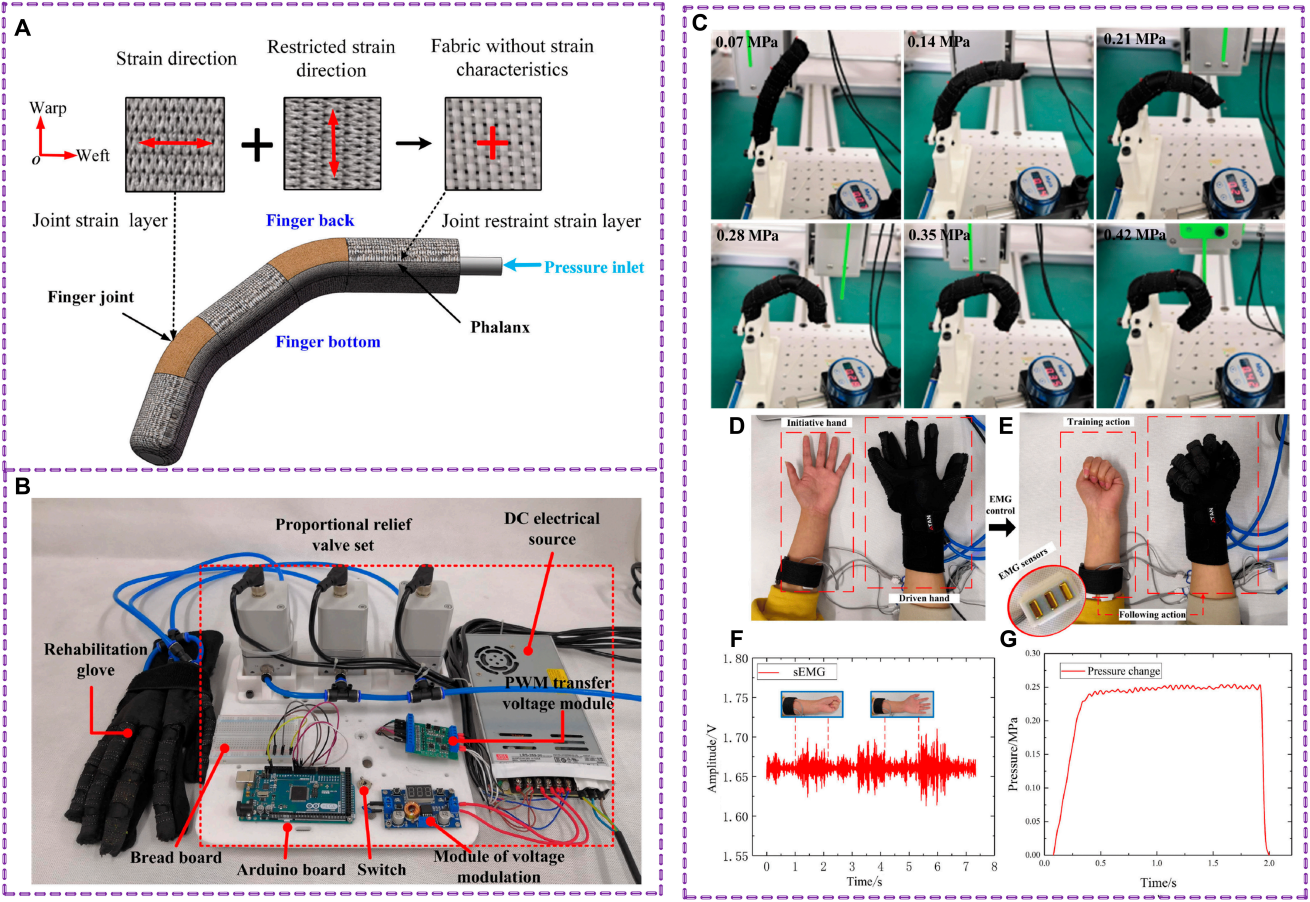
(A) Schematic diagram of the composition of the joint fabric reinforced soft body rehabilitation finger. (B) Control system of the soft rehabilitation glove. (C) Bending test for soft finger. (D) Passive training of hand stretching. (E) Passive training of hand stretching. (F) Continuous passive training EMG signal. (G) Pressure curve of periodic passive training. Reproduced with permission [120]. Copyright 2024, *IEEE Explore.*

A PD action tremor detection technique was presented by Sun et al. [117] to identify PD tremors during everyday activities. The wrist-worn accelerometers and gyroscope sensors were utilized to pick time- and frequency-domain hand-crafted characteristics from the dataset. To identify PD action tremors, these variables were then loaded into a variety of supervised ML models, including support vector machines (SVMs), KNNs, logistic regression (LR), and CNNs. Wang et al. [118] designed a wearable in-shoe monitoring system that featured a PC interface made with Python, a data processing module, and a flexible insole. Among the models that use various techniques, the artificial neural network (ANN) system has the best recognition accuracy. Using an Arduino microcontroller, Aliff et al. [119] have created a prototype smart rehabilitation hand device that uses an IoT and pneumatic system to operate a soft actuator for PD patients. The actuators are lightweight, simple, affordable, and safer than traditional ones, designed to match and support the range of a human finger. A new soft glove with a bionic method was designed by Chen et al. [120] using composite fabric material. Experiments show that the glove can achieve joint angles of 81°, 98°, and 72° under 0.42 MPa fluid pressure, producing torques of 1.18, 1.44, and 1.82 Nm, respectively. The glove also has a 30 times/min movement frequency for repetitive flexion/extension exercise (Fig. 9). The bionic glove’s grasping characteristics are dexterous, conforming to universal human hand grasping, and can assist daily life and rehabilitation needs.

ML algorithms can predict severe symptoms by analyzing IoT device data trends, enabling proactive treatment. By tailoring soft robotics operations to each patient’s unique needs, ML can enhance therapeutic efficacy, but their reliance on large amounts of high-quality data may pose a constraint [97,104,121]. Building and refining ML algorithms for reading complex biological data requires extensive experience and processing power, as sensor networks have evolved from single-channel to multi-channel and multi-mode, resulting in multi-dimensional, complex, and large sensor data [111]. Conventional data analysis techniques are insufficient for hydrogel flexible sensor networks, allowing medical professionals to create personalized treatment programs tailored to each patient’s specific requirements. The integration of sensors and actuators in a wireless body area network (WBAN) has gained popularity due to increased health awareness and the need for frequent updates [122]. IoT technology enables remote monitoring and adjustments, reducing the need for frequent clinical visits and providing ongoing support, thereby easing patients’ concerns [123].

### Virtual reality and augmented reality

In recent years, wearable optical technologies have gained a lot of interest [124]. The therapeutic potential of soft robotic devices will be determined by advancements in materials science, optics, electronics, sensors, haptic intelligence, and artificial intelligence (AI), with virtual reality (VR) and digital twins potentially aiding clinical translation. VR technology is being proposed as a superior rehabilitation tool, using patient data to create digital replicas that mimic the patient’s physical characteristics and the robotic system’s condition during treatments. This enhances feedback and control of soft robotic devices [125]. Near-eye displays that combine VR and AR to provide an immersive visual experience are 2 examples [126]. Actuators are required to provide these gadgets with features beyond just displaying pictures [127]. A digital patient, similar to a digital twin, creates a detailed map for personalized medicine [128]. However, physiological system disruptions and chaotic dynamics may hinder therapeutic applications due to the complexity of the patient’s digital copy.

Clinically validated VR/AR applications: For example, VR-based rehabilitation systems for stroke patients (e.g., the use of the VR mirror therapy approach) and VR exposure therapy for post-traumatic stress disorder (PTSD) have been supported by multiple randomized controlled trials (RCTs), demonstrating statistically significant improvements in patient outcomes compared to conventional methods. On the other hand, AR-assisted surgical navigation systems and VR-based empathy training tools largely remain in prototype or feasibility testing phases. These have shown promise in small-scale studies or simulations but lack large-scale RCT-based validation. Table 2 categorizes major VR/AR applications for PD based on their clinical validation level.

**Table 2.** Clinical validation of VR/AR applications in PD management

Application type	Technology used	Clinical validation status	Study type/trial phase	Sample size	Key outcomes	Ref.
Treadmill + VR visual cues	VR headset with a moving walkway	Validated	RCT	~30–50 patients	Improved gait speed, reduced freezing episodes	[158]
VR dual-task cognitive training	Immersive VR games for cognitive–motor control	Feasibility	Pilot trial	~20 patients	Enhanced cognitive–motor coordination	[159]
AR smart glasses (cueing)	Head-mounted AR device with visual cues	Proof-of-concept	Case series	<10 patients	Improved gait initiation in some users	[160–162]
AR home coaching app	Smartphone-based AR for exercise guidance	Proof-of-concept	Usability study	<10 participants	Good user engagement; clinical effects unknown	[163–165]

**Fig. 10.**
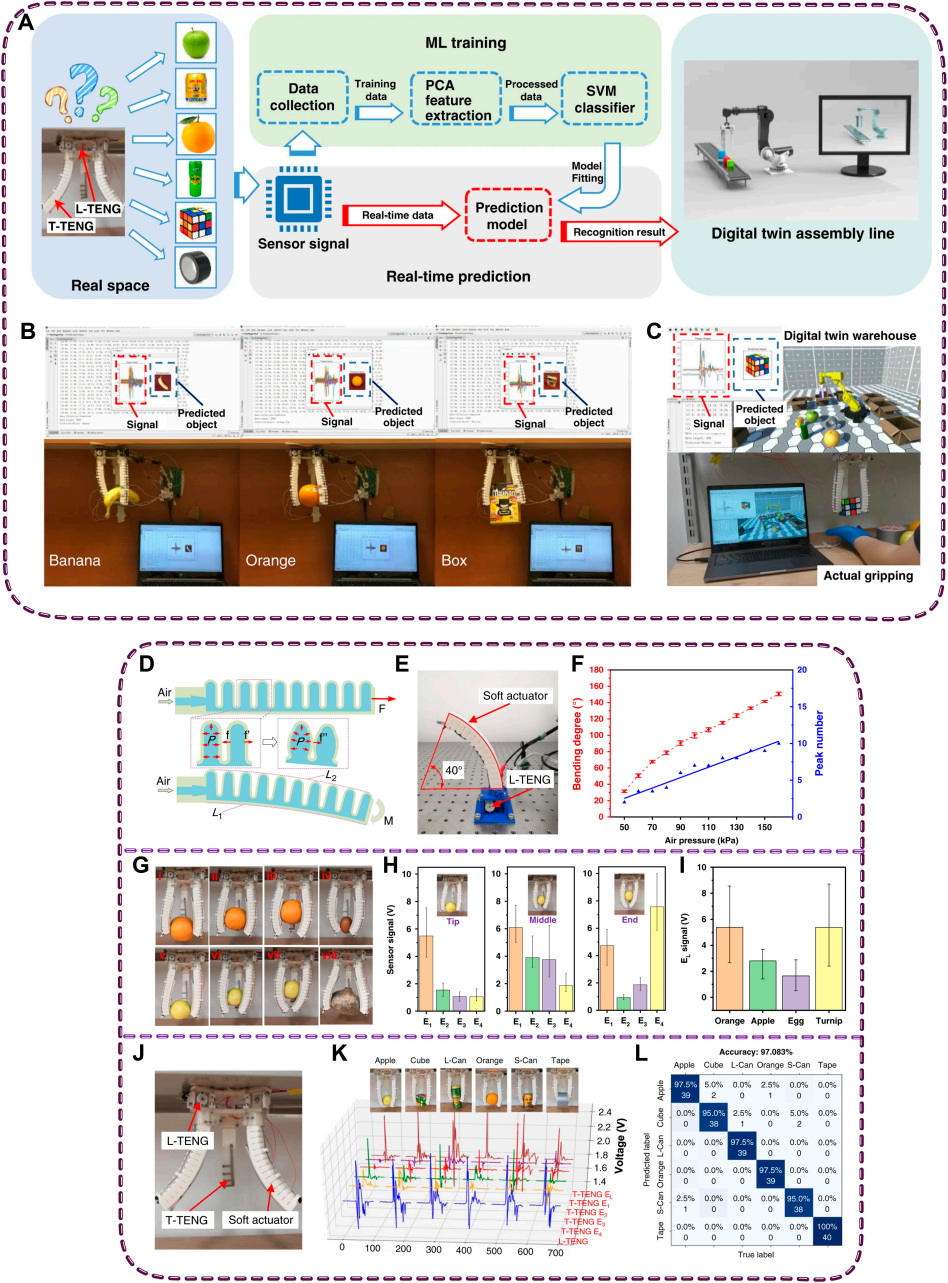
(A) In digital twin systems, such as digital twin production lines, the process proceeds from the gathering of sensory data to ML training and real-time prediction. To extract the data feature for support vector machine (SVM)-based ML training, principal components analysis (PCA) is used. (B) Method for predicting grabbed objects in real time. (C) The digital twin warehouse application and object identification were incorporated into the system interface. (D) Soft actuator’s mode of operation. (E) Setup of the apparatus while the length TENG (L-TENG) sensor is being characterized. (F) As air pressure increases, the L-TENG sensor’s output peak values are shown against the soft actuator’s bending degrees. (G) Diagrams showing how the gripper tests the T-TENG sensor by holding different items in different contact positions. (i to iii) Holding an orange in various ways, (iv) holding an egg, (v to vii) holding an apple in various ways, and (viii) holding a turnip. (H) Tactile TENG (T-TENG) sensor’s output signals for holding an apple in different contact positions. (I) The long electrode’s (E_L_) output when holding a variety of items with varying curvatures. (J) Gripper and sensor setup. (K) 3D graphs showing how the robotic sensor outputs react to various items, including tape, orange, long can (L-Can), cube, apple, and short can (S-Can). (L) ML result confusion map. Reproduced with permission from [132]. Copyright 2020, *Nature Communication.*

A new device called WeTac, developed by Yao et al. [129], is a skin-integrated haptic interface that uses electricity to produce tactile sensations. This device allows users to provide personalized feedback when interacting with virtual objects, enhancing human–machine interactions and addressing tactile information sensitivity challenges. A game-based, noninvasive health monitoring system for motor symptoms of PD was developed, collecting data from patients without reminding them of their medical condition. The system uses SVM to detect bradykinesia motor symptoms using a noninvasive approach [130]. The work by de Oliveira et al. [131] described the use of VR and serious games, along with wrist orthoses, to alleviate muscular stiffness in people with PD. The robotic wrist orthosis and modified Boccia game aid in rehabilitation through physical therapy and support the patient’s evolution analysis. Likewise, Jin et al. [132] developed a smart soft-robotic gripper system using triboelectric nanogenerator sensors to capture motion and tactile information (Fig. 10). The system uses distributed electrodes to detect contact position and area of external stimuli and a gear-based length sensor to detect elongation. The triboelectric sensory information is trained using an SVM algorithm to identify diverse objects with 98.1% accuracy. The system has been successfully used in virtual assembly lines and unmanned warehouse applications.

**Fig. 11.**
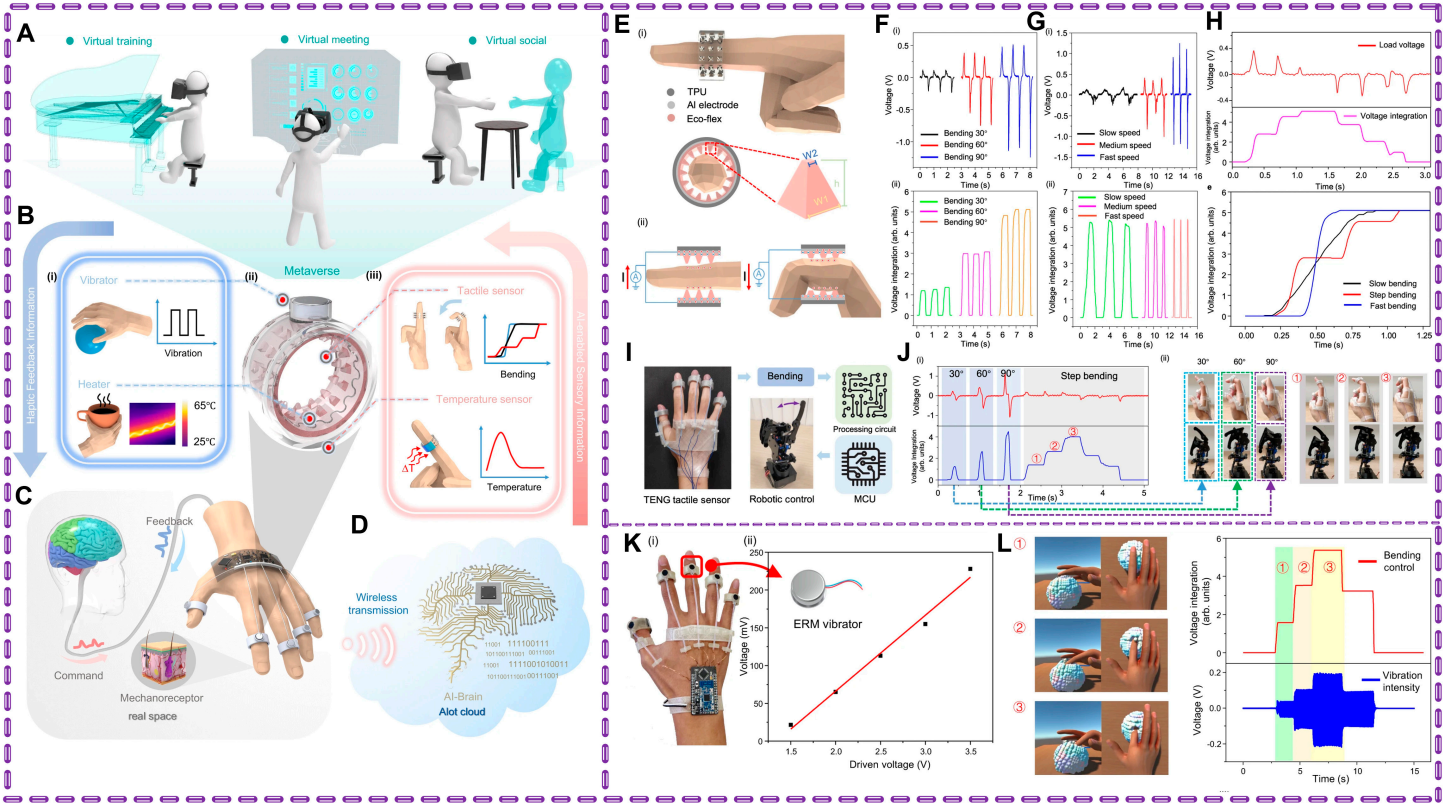
The intelligent system with multimodal sensing and feedback features is based on the ATH-Ring. (A) Visual representation of the metaverse, a virtual environment for enhanced social interaction, education, and enjoyment. (B) (i) The feedback capability is made possible by the heater and vibrator being combined. (ii) ATH-Ring’s intricate construction. (iii) Temperature and tactile sensors’ combined sensing capabilities. (C) Diagram of the organic brain network that underlies the real-world finger feeling experienced by humans. (D) AIoT cloud for processing and transmitting the gathered sensory data wirelessly. ATH-Ring characterization for continuous bending detection. (E) TENG tactile sensor’s (i) construction and (ii) operation. (F) For bending with varying angles and the same speed, the (i) load voltage and (ii) the matching integration value. (G) (i) load voltage and (ii) the corresponding integrated values for bending at different speeds, but at a constant angle of 90°. The tactile sensor for (H) step bending sensing and (E) continuous bending sensing has been verified. (I) The robotic collaborative operation system’s schematic. (J) The appropriate human and robotic finger motions are displayed in the (i) outputs of the TENG tactile sensor for continuous robotic finger bending control with the (ii) examples. IoT platform-compatible augmented multimodal haptic feedback system. (K) (i) Diagram showing the ATH-Ring-based HMI with eccentric rotating mass (ERM) vibrators integrated. (ii) Connection between the driving voltage of the vibrator and the vibration intensity measured by a PZT vibration sensor. The vibro-haptic feedback feature is demonstrated by (L) using one finger to squeeze a soft ball. Reproduced with permission [139]. Copyright 2022, *Nature Communications.*

Wearable technologies are gaining popularity due to their powerful, reliable, and AI-powered human–machine interfaces, which are increasingly used in mixed-media, digital, and physical contexts [133–136]. Zhang et al.’s [137] glove-based human–computer interaction (HMI) system makes use of polymer-based textile substrate metal electrode-triboelectric nanogenerators (PTSM-TENGs), to accomplish the tasks of gesture visualization and robot hand control. Integrating conductive hydrogels with flexible electronics might pave the way for the advancement of wearable technology, AI, healthcare monitoring, and soft robotics [66]. This even offers fresh perspectives on HMIs, self-feedback intelligent soft robotics, and enhanced somatosensory materials [136,138]. Wearable wireless gait monitoring devices can improve the quality of life for individuals with PD by preventing falls and detecting freezing. These devices provide biofeedback, such as voice alarms and instructions, to aid rehabilitation. They continuously gather data while walking, and if unusual gait patterns or issues are detected, they provide verbal feedback through headphones. Sun et al. [139] have developed augmented tactile perception and haptic feedback rings called ATH-Ring with multimodal sensing and feedback capabilities (Fig. 11). These rings consist of triboelectric and pyroelectric sensors for tactile and temperature perception and vibrators and nichrome heaters for vibro- and thermo-haptic feedback. The ring can be driven by a low-power wireless platform for wearable/portable scenarios. This fusion of sensing and feedback creates an interactive metaverse platform with cross-space perception capabilities.

Although this study describes new developments in AR, hydrogel-based soft robotics, and AI for the treatment of PD, it is crucial to acknowledge that many of these systems still lack clinical validation. Currently, few technologies—like VR-based gait training—have completed RCTs, and most research focuses on system design, material characterization, or short-term viability. On the other hand, AI-driven adaptive support systems and wearable robotics with hydrogel actuators are still mostly in the preclinical or proof-of-concept phase. To prove safety, effectiveness, and long-term effects, longitudinal clinical studies that include patient-reported outcomes and practical usability evaluations are essential. To guarantee that new technologies can be appropriately and successfully incorporated into standard clinical practice, it is imperative that this translational gap be filled.

## Current Challenges

The soft robotic system for PD patients is being tested for its effectiveness in improving motor function and tremors. Although the combination of IoT, AI, and hydrogel-based soft robotics has great potential for the treatment of PD, there are still a number of important obstacles to overcome. Since wearable IoT devices and cloud-based algorithms exchange data continuously, there is a risk of hacks and illegal access, making data privacy and security crucial. To reduce these concerns, strong encryption mechanisms and user permission procedures must be put in place. Furthermore, further research is needed to determine how long hydrogel-based materials will last, particularly when subjected to repetitive mechanical strain, temperature changes, and extended skin contact. Degradation of the equipment without showing resilience may eventually jeopardize patient safety and functionality. Lastly, access to these cutting-edge systems may be restricted by socioeconomic restrictions. Widespread adoption may be hampered by high production costs, inadequate insurance coverage, and differences in computer competence, especially among underprivileged or elderly persons. To guarantee fair access and practical effect, these problems must be addressed through inclusive design, scalable manufacturing, and policy support.

Prioritizing patient safety and ethical concerns, the system undergoes rigorous scientific assessment through long-term follow-up and participant feedback. The ultimate goal is to enhance the system’s effectiveness in assisting PD patients [12]. The testing process involves a baseline assessment of the participant’s health and motor abilities, conducted in regulated environments, and continuous observation to assess their behavior and system performance [140]. Hydrogel-based wearable soft robotics have the potential for treating PD, but they face challenges such as mechanical durability, integration with electrical components, and maintaining consistent operation under variable environmental conditions. These biocompatible and flexible materials are susceptible to deterioration, especially in repeated motions. It is crucial to ensure their integrity for extended usage and ensure that they are comfortable and secure for each patient’s anatomy without irritating them or triggering allergic responses [141].

The integration of IoT and ML technology into wearable soft robots for PD therapy faces several challenges. Data security and privacy are crucial, as IoT devices collect and send vital health data. Network dependability is vital, as loss could cause delays in treatment delivery and patient safety. High power needs and interoperability with other devices and healthcare systems are also significant. IoT system complexity also makes maintenance and troubleshooting difficult, requiring specific knowledge from users and healthcare practitioners [96,120]. Training accurate ML models requires large, diverse datasets, which can be challenging due to the significant variation in symptoms and illness development among patients. ML algorithms must be resilient to handle noisy and missing data from wearables, and real-time processing capabilities are crucial for treatment efficacy. Clinical acceptability depends on interpretability, and medical professionals must trust the decision-making processes of ML models. Integrating ML into wearable robots requires careful consideration of computational resources and energy usage, as devices must be capable of continuous operation without regular recharging.

## Conclusion and Future Work

**Fig. 12.**
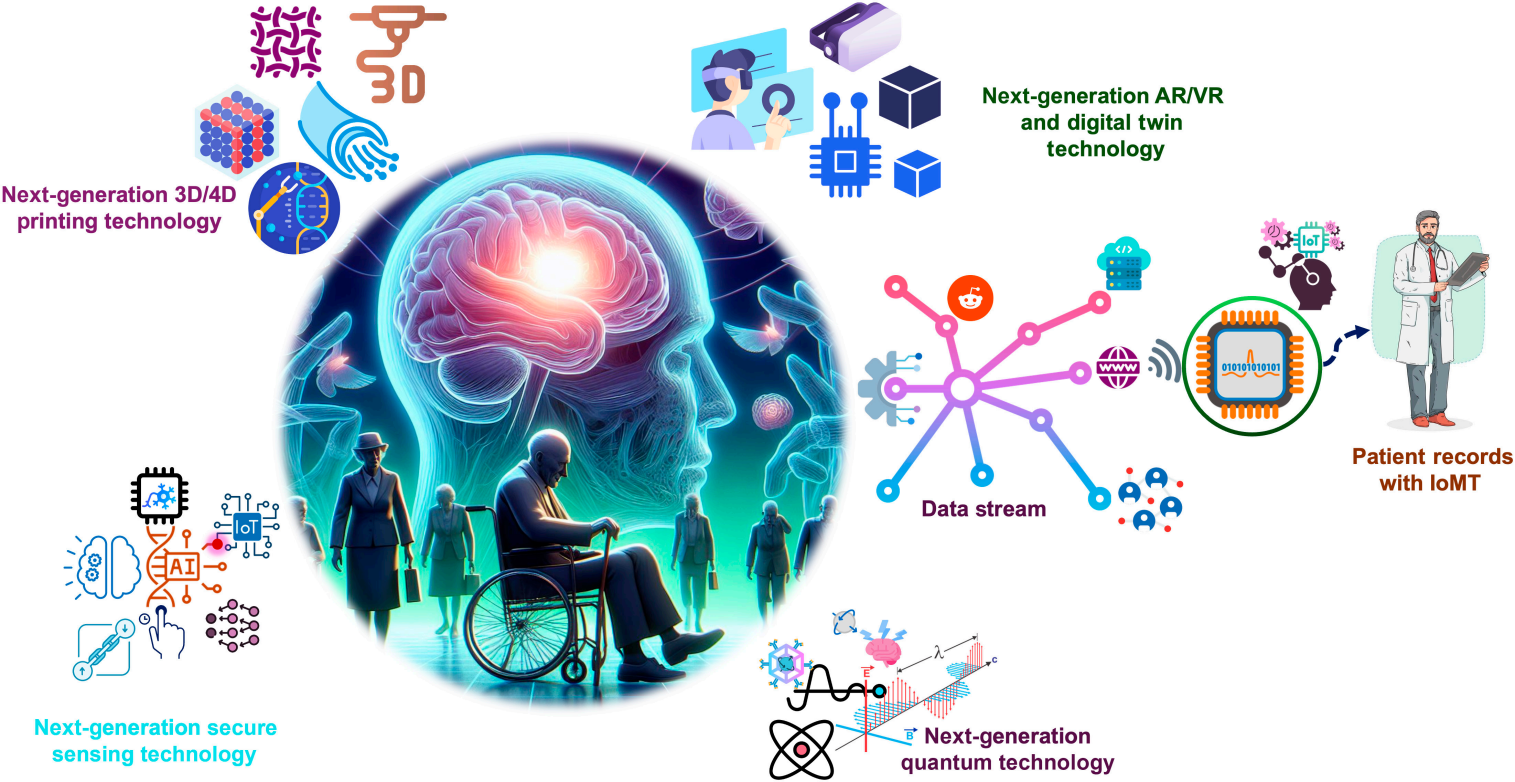
A futuristic ecosystem integrating next-generation technologies for personalized healthcare: 3D/4D printing for advanced prosthetics, secure sensing for patient monitoring, AR/VR with digital twins for immersive diagnostics, IoT-enabled patient records, and quantum technology for data processing for PD.

The integration of hydrogel-based soft robotics, AI, and AR presents a promising approach for managing PD. These next-generation systems provide targeted motor assistance and real-time monitoring, enhancing patient autonomy, functional capacity, and overall quality of life. Embedded IoT sensors and ML algorithms enable continuous adaptation to individual symptom profiles, supporting personalized, responsive care [80,120,142,143]. Preliminary evaluations suggest high patient satisfaction with the comfort and usability of hydrogel-based devices, which are lightweight, ergonomic, and compatible with long-term use. The potential for seamless integration into daily routines promotes adherence and supports a shift toward home-based rehabilitation. Future studies (as illustrated in Fig. 12) may include improved sensor technologies, smaller components, and sophisticated materials [144,145]. The next generation of wearable robots should use human–robot interface strategies, including multimodal fusion, human-in-the-loop control, neuromuscular interface, flexible electronics, and biomechatronic chips [146]. Additional ML algorithms are needed to improve the system’s ability to adjust in real time to each patient’s unique needs and condition [12].

As the field evolves, ongoing improvements in sensor miniaturization, material sophistication, and control strategies, such as human-in-the-loop and multimodal fusion, will be essential. Further work should prioritize long-term clinical validation, including RCTs, patient-reported outcomes, and engagement with community stakeholders to align technological advances with user needs.
